# *Enterobacter cloacae* inhibits human norovirus infectivity in gnotobiotic pigs

**DOI:** 10.1038/srep25017

**Published:** 2016-04-26

**Authors:** Shaohua Lei, Helen Samuel, Erica Twitchell, Tammy Bui, Ashwin Ramesh, Ke Wen, Mariah Weiss, Guohua Li, Xingdong Yang, Xi Jiang, Lijuan Yuan

**Affiliations:** 1 Department of Biomedical Sciences and Pathobiology, Virginia-Maryland College of Veterinary Medicine, Virginia Tech, Blacksburg, VA 24061, USA; 2 Division of Infectious Diseases, Cincinnati Children’s Hospital Medical Center, Cincinnati, OH 45229, USA

## Abstract

Human noroviruses (HuNoVs) are the leading cause of epidemic gastroenteritis worldwide. Study of HuNoV biology has been hampered by the lack of an efficient cell culture system. Recently, enteric commensal bacteria *Enterobacter cloacae* has been recognized as a helper in HuNoV infection of B cells *in vitro*. To test the influences of *E. cloacae* on HuNoV infectivity and to determine whether HuNoV infects B cells *in vivo*, we colonized gnotobiotic pigs with *E. cloacae* and inoculated pigs with 2.74 × 10^4^ genome copies of HuNoV. Compared to control pigs, reduced HuNoV shedding was observed in *E. cloacae* colonized pigs, characterized by significantly shorter duration of shedding in post-inoculation day 10 subgroup and lower cumulative shedding and peak shedding in individual pigs. Colonization of *E. cloacae* also reduced HuNoV titers in intestinal tissues and in blood. In both control and *E. cloacae* colonized pigs, HuNoV infection of enterocytes was confirmed, however infection of B cells was not observed in ileum, and the entire lamina propria in sections of duodenum, jejunum, and ileum were HuNoV-negative. In summary, *E. cloacae* inhibited HuNoV infectivity, and B cells were not a target cell type for HuNoV in gnotobiotic pigs, with or without *E. cloacae* colonization.

Human noroviruses (HuNoVs), non-enveloped positive-strand RNA viruses, are the leading cause of viral epidemic acute gastroenteritis worldwide^1^. HuNoVs infect people of all ages, the gastroenteritis is characteristically self-limiting with a duration of 1 to 3 days, but it can be severe and prolonged in infants, young children, elderly, and immunocompromised individuals^2^. As members of the *Norovirus* genus in the *Caliciviridae* family, noroviruses are divided into six genogroups (GI - GVI) based on viral capsid gene sequences, but only GI, GII, and GIV are found in humans and thus known as HuNoVs^3^. Although at least 32 different HuNoV genotypes have been further classified^4^, genogroup II genotype 4 (GII.4) has been the predominant genotype causing global acute gastroenteritis outbreaks^5^. In the past two decades, six major epidemics have occurred due to novel GII.4 variants that evolved by recombination and mutation, including the most recent strain, GII.4 Sydney_2012^6^. During the season of 2014–2015, newly emerging GII.17 variants caused outbreaks in Asia, and the urgent need to control the global spread of GII.17 has gained recent attention^7^,^8^,^9^. Unfortunately, no vaccines or virus-specific therapies are currently available to prevent or treat HuNoV infection^10^.

HuNoV research has long been impeded by the lack of a robust cultivation system and a suitable animal model. Limited knowledge of HuNoV biology are mainly from viral infection studies in chimpanzees^11^, gnotobiotic (Gn) calves and pigs^12^,^13^,^14^,^15^, immunodeficient mice^16^, and human volunteers^2^. *Enterobacter cloacae* was screened from commensal enteric bacteria with surface histo-blood group antigen (HBGA) expression and the ability to bind to HuNoV specifically^17^. *E. cloacae* was subsequently found to promote HuNoV infection of human B cells (BJAB cell line) *in vitro*^18^, which is a novel HuNoV infection system that redefined the range of HuNoV cell tropism and viral infection factors. In this cell culture model, B cells supplemented with free HBGA or HBGA-expressing enteric bacteria, such as *E. cloacae*, were susceptible to HuNoV infection^19^. However, there is a total lack of *in vivo* studies to support the role of *E. cloacae* or other HBGA-expressing enteric bacteria in enhancing HuNoV infection of B cells. In addition, the low-level viral replication in such cultured B cells was inconsistent with high-level virus shedding in human patients, suggesting that B cells might not be the major target cell type of HuNoV^20^. Therefore, *in vivo* evidence is essential to test the stimulatory role of *E. cloacae* and to confirm that B cells are a natural target for robust HuNoV infection and replication.

The neonatal Gn pigs recapitulate the hallmark features of the gastrointestinal tract in young children and have been widely used for enteric virus infection^21^,^22^. HuNoV pathogenesis studies and vaccine evaluations in Gn pigs have high translational relevance to those of humans^13^,^14^,^23^. Compared to chimpanzees (no longer available for biomedical research) and immunodeficient mice, Gn pigs are currently preferable for HuNoV infection study in many ways, such as the ability to become infected via oral route and resulting in diarrhea and fecal virus shedding^24^. In addition, the germ-free environment in the Gn system has enabled the studies of interaction between virus and specific bacterial strains^25^,^26^,^27^, as well as human microbiota^28^,^29^. In this study, via *E. cloacae* colonization in the well-established neonatal Gn pig model of HuNoV infection and diarrhea, we aimed to (i) elucidate the effects of *E. cloacae* on HuNoV infectivity *in vivo*, (ii) determine whether HuNoV infects B cells *in vivo*, and (iii) explore the mechanism of altered HuNoV infectivity in the presence of *E. cloacae*.

## Results

### *E. cloacae* reduced HuNoV shedding but not diarrhea

**Figure 1:**
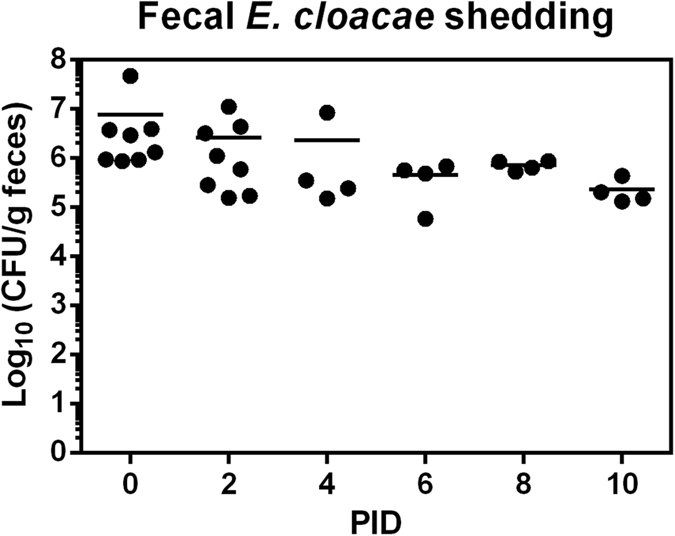
Fecal *E. cloacae* shedding. Colonization of *E. cloacae* in the Gn pigs throughout the HuNoV infection study was confirmed by monitoring fecal shedding. The colony forming unit (CFU) was measured in serial dilutions of fecal samples by enumeration of colonies grown on agar plates of culture media. Data shown are pooled from independent experiments performed on each day, with individual animal data points.

To evaluate the effects of *E. cloacae* on HuNoV infection and diseases *in vivo*, a group of Gn pigs were inoculated with three doses of *E. cloacae* on 3, 4, and 5 days of age. Together with another group of control pigs, all were inoculated with 2.74 × 10^4^ genome copies of HuNoV GII.4/2006b at 6 days of age, which was post inoculation day 0 (PID0), then euthanized on PID3, PID7, or PID10. To confirm the colonization of *E. cloacae* in these Gn pigs, fecal *E. cloacae* shedding was determined daily from PID0 to euthanasia day. *E. cloacae* was detected in all treated pigs (Fig. 1), whereas the control pigs remained sterile throughout this study (data not shown).

**Figure 2:**
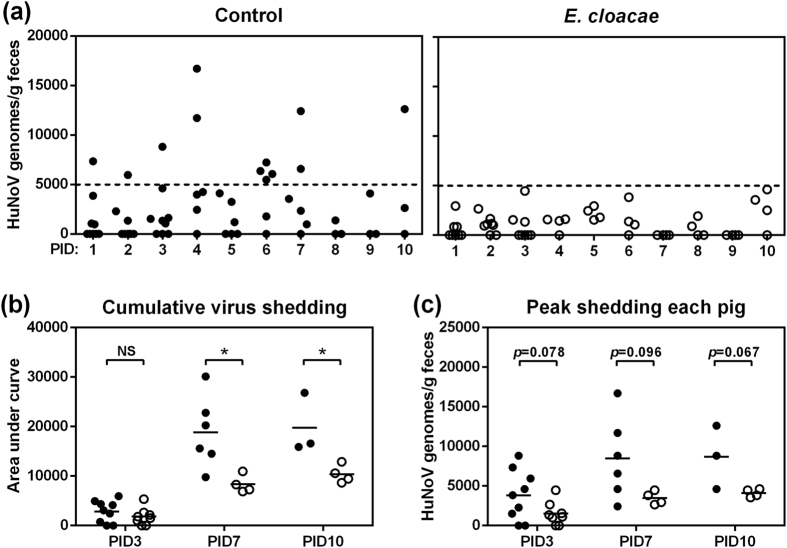
Lower HuNoV shedding in *E. cloacae* colonized Gn pigs. (**a**) Fecal HuNoV shedding was monitored daily from PID1 to PID10 by qRT-PCR to quantify viral genomes in feces. Dashed line indicates 5000 HuNoV genomes. (**b**) Cumulative virus shedding was shown as area under curve calculated for individual pigs based on (**a**). (**c**) Peak shedding titers from PID1 to PID3, PID7, or PID10 in each pig was presented. Sample sizes are shown in Table 1. Data are presented as mean with individual animal data points (**b**,**c**). Statistical significance was determined by Student’s *t*-test. NS, not significant, *P < 0.05.

Fecal HuNoV shedding was monitored daily in both control and *E. cloacae* colonized Gn pigs after viral inoculation (Fig. 2a). In *E. cloacae* colonized pigs, virus shedding was clearly lower than that of control, as the shedding titers for all pigs on each day were below 5000 genome copies per gram of feces. The incidence of virus shedding showed that compared to control pigs, *E. cloacae* colonized pigs had a significantly shorter mean duration in PID10 subgroup (6.7 versus 5.5 days) (Table 1). To further characterize the shedding titers, cumulative and peak shedding for individual pigs were calculated. The cumulative virus shedding in *E. cloacae* colonized pigs was significantly lower on PID7 and PID10 (Fig. 1b), and there was also a trend for lower peak shedding titers in *E. cloacae* colonized pigs (Fig. 1c). No significant difference in the incidence of diarrhea was observed among groups at any time points (Table 1), indicating *E. cloacae* colonization did not affect the incidence of diarrhea in Gn pigs after HuNoV infection. These data demonstrated that *E. cloacae* colonization inhibited HuNoV shedding in Gn pigs.

### *E. cloacae* reduced HuNoV titers in intestinal tissues and blood

**Figure 3:**
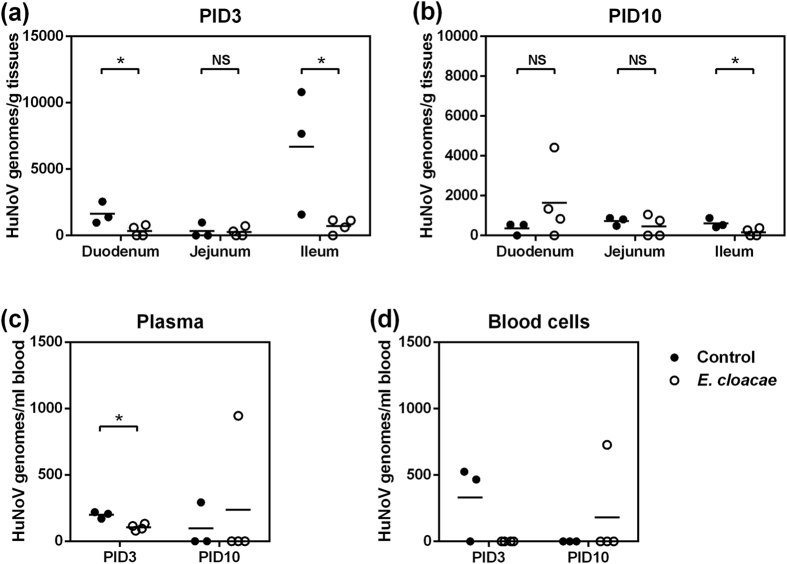
Lower HuNoV titers in small intestine and blood in *E. cloacae* colonized Gn pigs. HuNoV genomes in duodenum, jejunum, and ileum in pigs euthanized on PID3 (**a**) and PID10 (**b**) were measured by qRT-PCR. HuNoV genomes in plasma (**c**) and whole blood cells (**d**) were measured by qRT-PCR. Sample sizes in control groups, PID3 *n* = 3, PID10 *n* = 3; in *E. cloacae* groups, PID3 *n* = 4, PID10 *n* = 4. Data are presented as mean with individual animal data points. Statistical significance was determined by Student’s *t*-test. NS, not significant, *P < 0.05.

Previous studies showed that HuNoV (GII.4) antigen was observed in duodenum and jejunum of Gn pigs^13^,^14^,^15^. In this study, analysis of the tissue samples confirmed the existence of HuNoV genomes in duodenum and jejunum, and viral genomes were also detectable in ileum for both control and *E. cloacae* colonized pigs (Fig. 3a,b). Compared to control pigs, on PID3, virus titers were significantly lower in duodenum and ileum of *E. cloacae* colonized pigs (Fig. 3a). On PID10, virus titer was significantly lower in ileum of *E. cloacae* colonized pigs (Fig. 3b).

Previously, HuNoV was detected in blood in humans with gastroenteritis^30^,^31^, as well as in Gn calves and pigs after HuNoV challenge^12^,^13^. In this study, viral genomes were present in plasma in 4 of 6 control pigs (66.7%) and 5 of 8 (62.5%) *E. cloacae* colonized pigs, and virus titer on PID3 was significantly lower in *E. cloacae* colonized pigs (Fig. 3c). In addition, viral genomes were also detectable in whole blood cells in 2 of 6 control pigs (33.3%) and in 1 of 8 *E. cloacae* colonized pigs (12.5%) (Fig. 3d). Taken together, the reduced HuNoV titers in intestinal tissues and in blood indicated the inhibitive role of *E. cloacae* for HuNoV infection in Gn pigs.

### HuNoV antigen was observed in enterocytes but not in B cells

**Figure 4:**
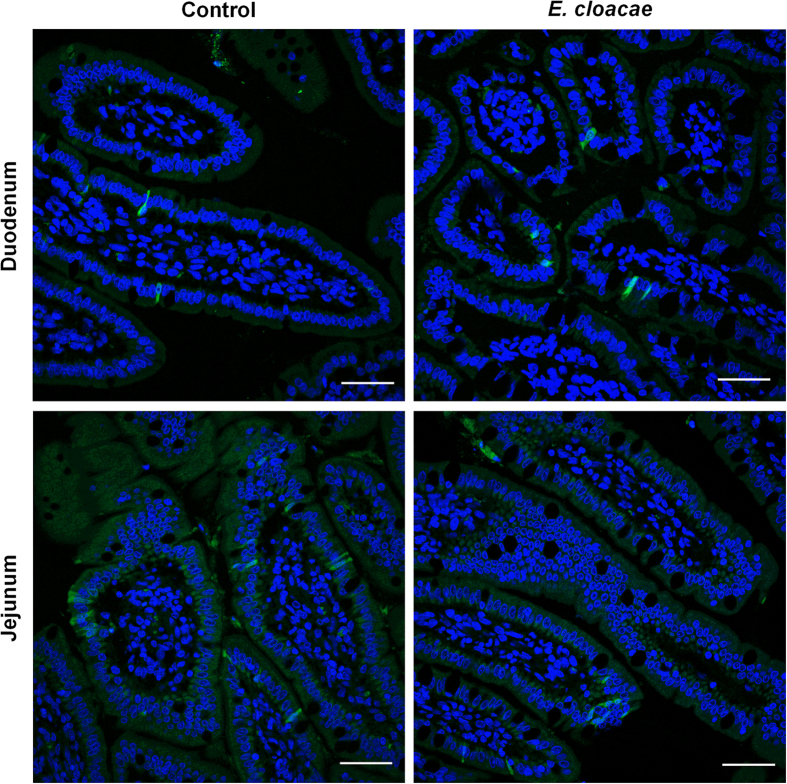
HuNoV infection of enterocytes in Gn pigs. HuNoV capsid in duodenum (top panel) and jejunum (bottom panel) of Gn pigs euthanized on PID3 was detected by immunohistochemistry. Cell nuclei (blue) were counterstained with HuNoV capsid (bright green). Representative images showing HuNoV was detected in enterocytes in control and *E. cloacae* colonized Gn pigs. Scale bar, 50 μm.

To confirm HuNoV infection of enterocytes in Gn pigs and to identify virus-infected cells in *E. cloacae* colonized pigs, immunohistochemistry (IHC) was performed to detect the viral capsid protein VP1 on sections of small intestine from both groups. As expected, HuNoV antigen was observed in enterocytes of duodenum and jejunum from control pigs. In *E. cloacae* colonized pigs, both duodenal and jejunal enterocytes were also the only positive cells, whereas cells of the lamina propria were negative for both groups (Fig. 4).

**Figure 5:**
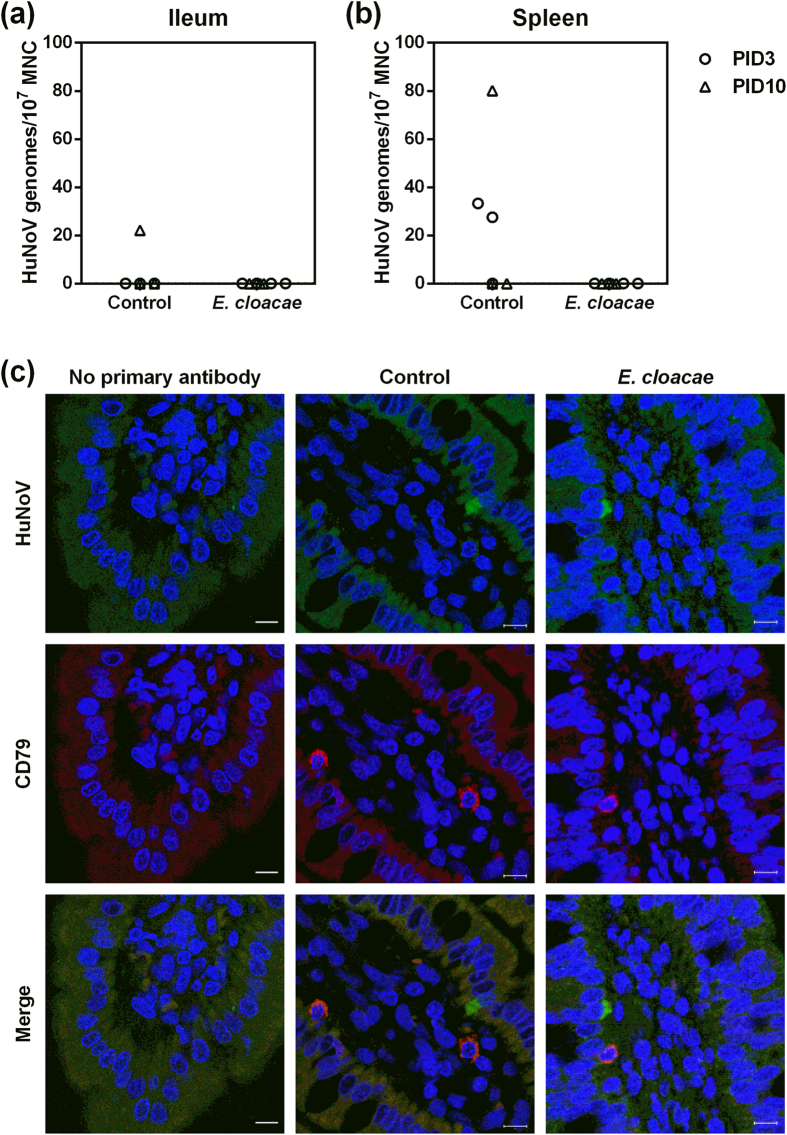
HuNoV infection of B cells was not observed in Gn pigs. MNC from ileum (**a**) and spleen (**b**) were isolated and measured for HuNoV titers by qRT-PCR. Data are presented as individual animal data points. (**c**) Ileum tissue sections were stained to detect HuNoV capsid protein (bright green) and B cells (CD79^+^, red) with counterstain of cell nuclei (DAPI, blue). Representative images showing that HuNoV was observed in enterocytes but not in B cells in control and *E. cloacae* colonized Gn pigs. Scale bar, 10 μm.

To determine whether HuNoV infects B cells in the presence of *E. cloacae* in Gn pigs, we isolated total mononuclear cells (MNC) from ileum and spleen, then performed qRT-PCR to detect viral genomes. 1 of 6 control pigs had detectable viral genomes in ileal MNC (Fig. 5a), and 3 of 6 control pigs had detectable viral genomes in splenic MNC (Fig. 5b), although the titers were as low as 20 genomic copies in 10^7^ MNC. However, no viral genomes were detected in ileal or splenic MNC in *E. cloacae* colonized pigs, presumably resulting from the lower HuNoV infection in these pigs. Furthermore, IHC for both viral antigen and B cells was performed in ileum, because ileum is populated with B cells in conventional and Gn pigs^32^, and viral genomes were detected in ileum (Fig. 3a). B cells were observed in the lamina propria, as probed by primary antibody targeting cellular marker CD79, while HuNoV antigen was observed only in enterocytes. Thus, there was no signal co-localization in sections from control and *E. cloacae* colonized pigs, even though B cells could be located in close proximity to HuNoV positive cells (Fig. 5c). In all, these data suggest that B cells are not a HuNoV target in Gn pigs, with or without *E. cloacae* colonization.

### *E. cloacae* promoted gut immunity

**Figure 6:**
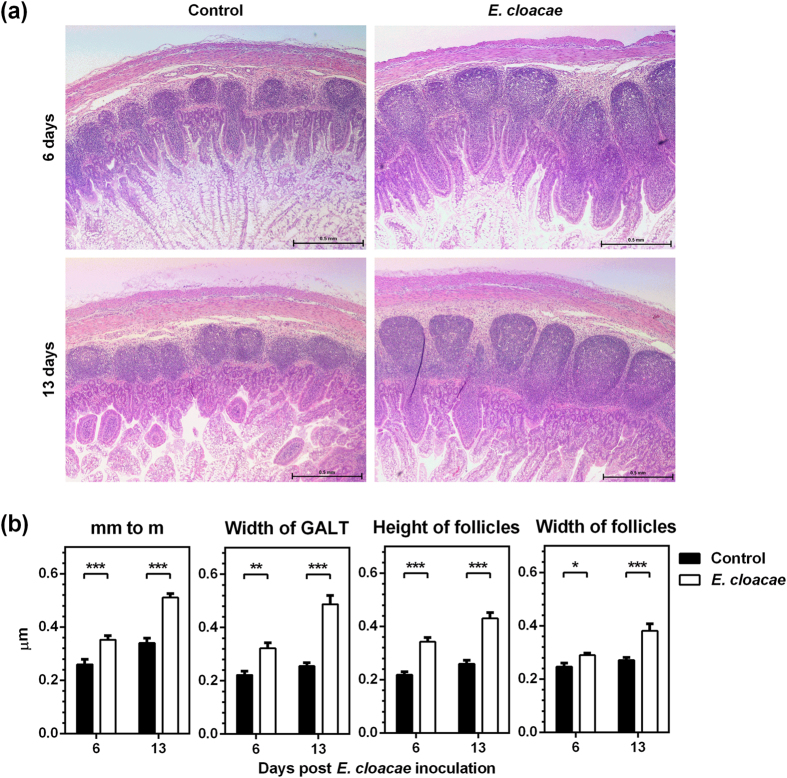
Colonization of *E. cloacae* in Gn pigs stimulated the development of IPP. (**a**) Representative H&E staining images of ileum showing more developed IPP in *E. cloacae* colonized Gn pigs compared to that of control pigs on both 6 days (top panel) and 13 days (bottom panel) post *E. cloacae* inoculation, Scale bar, 0.5 mm. (**b**) 12 random locations including all pigs in each group were measured to characterize the IPP size, including width from muscularis mucosae to muscularis (mm to m) associated with gut associated lymphoid tissue (GALT), width of GALT, height and width of follicles. Statistical significance was determined by Mann-Whitney test. *P < 0.05; **P < 0.01; ***P < 0.001. Error bars denote SEM.

Probiotic lactobacilli colonization enhanced gut immunity in Gn pigs by promoting intestinal T cell and B cell responses, as well as the secretion of intestinal immunoglobulins^25^,^26^. Lactobacilli protected against rotavirus diarrhea and also functioned as adjuvants for rotavirus vaccine by promoting protective immune responses^27^,^33^,^34^. We hypothesized that *E. cloacae* colonization might promote gut immunity in Gn pigs as well, which in return would inhibit HuNoV infectivity. To determine the role of *E. cloacae* in intestinal immune system development, we compared the sizes of ileal Peyer’s patches (IPP) for both groups. In *E. cloacae* colonized pigs, IPP were significantly larger and more developed than those of control pigs on 6 days and 13 days post inoculation of *E. cloacae* (Fig. 6a). The IPP in *E. cloacae* colonized pigs were characterized by significantly greater width from muscularis mucosae to muscularis associated with gut associated lymphoid tissue (GALT), width of GALT, height and width of follicles (Fig. 6b).

## Discussion

The lack of a robust cell culture and animal model for HuNoVs propagation has long eluded study of HuNoV biology and development of antiviral therapies. Among the *in vitro* models, HuNoVs reverse genetics systems containing subgenomic or genomic RNA have been established^35^,^36^, but the virus-like particle or virion production was inefficient. Reports of HuNoVs infection and replication observed in 3D intestinal epithelial cells were not reproducible^37^,^38^. Although HuNoV infection has been observed in chimpanzees^11^, the species is no longer available for the entire biomedical research due to ethical concerns. The BALB/c Rag/ɣc^−/−^ mouse was recognized as a HuNoV infection model via intraperitoneal inoculation^16^, but it is not suitable for HuNoV propagation due to the low robustness of replication and the lack of virus shedding. Given the limitations of current models, clinical fecal samples containing HuNoV from patients have been the only resource for HuNoV infection studies. Recently, HuNoV cultivation was described in human B cells (BJAB cell line) with free HBGA or HBGA-expressing *E. cloacae* supplementation^18^,^19^. Hence, we expanded our Gn pig model with *E. cloacae* colonization, which should enhance HuNoV infection and replication. Our original objective was to develop a pig model for effective HuNoV propagation. Surprisingly, *E. cloacae* inhibited HuNoV infectivity in Gn pigs instead.

First, fecal virus shedding has been used as direct evidence to characterize the status of HuNoV infection *in vivo*. Our data showed that the incidence of shedding and the shedding amount in *E. cloacae* colonized pigs were less than those of control pigs, including lower cumulative shedding and peak shedding in individual pigs (Table 1 and Fig. 2). Second, the small intestine has been shown to be the primary HuNoV infection site in Gn pigs^13^,^14^,^15^. We also confirmed the existence of viral genomes in all sections of small intestine in both groups, and the titers were significantly lower in *E. cloacae* colonized pigs. In addition, lower viremia was observed in *E. cloacae* colonized pigs (Fig. 3). As HuNoV genomes were present in whole blood cells, it is likely that those were virions captured by phagocytes and translocated through blood circulation, since ileal and splenic MNC had detectable viral genomes as well. Third, *E. cloacae* was expected to facilitate HuNoV infection of B cells based on the *in vitro* model, but no HuNoV-positive B cells were observed in ileum sections even though the adjacent enterocytes were HuNoV positive. In addition, the entire lamina propria in sections of duodenum, jejunum, and ileum were HuNoV-negative, in both control and *E. cloacae* colonized pigs.

Enteric bacteria can bind to HuNoV via surface HBGA^17^. It is likely that HBGA-expressing *E. cloacae* serves as a blockade between HuNoV and target cells *in vivo* instead of facilitating virion attachment, and thus leads to lower rates of infection. Both inhibition and enhancement of HuNoV P particles attachment via such binding have been observed *in vitro* for other probiotic bacteria, such as *Lactobacillus casei* BL23 and *Escherichia coli* Nissle 1917^39^. Our data suggested that inhibition of attachment by *E. cloacae* was the scenario in pigs *in vivo*, resulting in decreased HuNoV infectivity. Another reason for the lower HuNoV infectivity might be due to the enhanced development of gut immunity by *E. cloacae* colonization. We observed that colonization of *E. cloacae* in Gn pigs stimulated the development of IPP. Further and longer term studies will be required to determine the magnitude of immune responses to HuNoV infection between the two groups.

The binding between *E. cloacae* and HuNoV may also play a role in virus retention *in vivo*. We observed that virus titer in duodenum decreased from PID3 to PID10 in control pigs, but it appeared to be unchanged or even increased in *E. cloacae* colonized pigs (Fig. 3a,b). *E. cloacae* is naturally resistant to broad-spectrum antibiotics^40^, thus it could be dominant in gut microbiota in immunocompromised patients, who also require antibiotic therapy to manage microbial infections in many cases^41^. Increased *E. cloacae* might in return contribute to persistence of HuNoV infection in immunocompromised hosts by virus retention.

In summary, colonization of *E. cloacae* in neonatal Gn pigs inhibited HuNoV infectivity, including reduced virus shedding, lower viral genome titers in intestinal tissues and in blood. HuNoV infection of B cells was not observed in duodenum, jejunum, or ileum in either control or *E. cloacae* colonized pigs. To our knowledge, this is the first *in vivo* study to evaluate the effects of *E. cloacae* on HuNoV infectivity, and our study paves the way for future studies of the interaction between HuNoV and enteric bacteria *in vivo*.

## Materials and Methods

### Virus and bacterium

A pool of stool containing GII.4/2006b variant 092895 (GenBank accession no. KC990829) was collected in 2008 by Dr. Xi Jiang’s laboratory at Cincinnati Children’s Hospital Medical Center from a child with HuNoV gastroenteritis. Stool sample collection was conducted in accordance with protocols approved by the institutional review boards of the Cincinnati Children’s Hospital Medical Center (IRB number: 2008-1131), and informed consent was obtained from parents or child. The stool was processed as inoculum and stored in our laboratory for studies of HuNoV infection in Gn pigs^14^. *Enterobacter cloacae* was purchased from ATCC (ATCC 13047) and grown in nutrient broth overnight at 30 °C with shaking at 250 rpm. Overnight cultures containing 15% glycerol were stored in −80 °C freezer for colonization of Gn pigs. The final concentration of *E. cloacae* was measured in serial dilutions by enumeration of colony forming unit grown on nutrient broth agar plates.

### *E. cloacae* colonization and HuNoV inoculation of Gn pigs

Near-term Yorkshire cross breed pigs were derived by hysterectomy and maintained in Gn pig isolators as described previously^42^. A subset of pigs was orally inoculated with 10^4^ CFU of *E. cloacae* daily on 3, 4, and 5 days of age to initiate colonization, which was monitored via testing fecal shedding. Control pigs received a diluent only. All pigs were orally inoculated with 2.74 × 10^4^ viral RNA copies of HuNoV at 6 days of age. Four ml of 200 mM sodium bicarbonate was given 15 min prior to HuNoV inoculation to reduce gastric acidity. Pigs were euthanized on PID3, PID7, or PID10 for collection of blood, intestinal contents, and tissues. Forty cm of distal ileum and whole spleen organ were collected for the isolation of MNC^22^. Pigs used in this study were HBGA-typed to be A^+^ and/or H^+^, and sterility was confirmed one day before *E. cloacae* inoculation for all pigs, as well as on euthanasia day for control pigs, as previously described^14^. All animal experimental procedures were conducted in accordance with protocols approved by the Institutional Animal Care and Use Committee at Virginia Tech (IACUC protocol: 14-108-CVM).

### Assessment of diarrhea and HuNoV shedding

Pig feces were collected daily by rectal swabs following HuNoV inoculation to assess diarrhea and fecal virus shedding. Fecal consistency was scored as follows: 0, solid; 1, semisolid; 2, pasty; 3, semiliquid; and 4, liquid. Pigs with daily fecal scores of 2 or greater were considered diarrheic. Virus shedding was determined as described previously^14^. Briefly, pig feces on rectal swabs were released by swirling in 1 ml PBS and processed as solution, 250 μl of which was prepared for total RNA isolation using TRIzol LS (Thermo Fisher Scientific). The RNA pellet was resuspended in 40 μl RNase-free water, and 5 μl RNA was used in a TaqMan qRT-PCR reaction to detect HuNoV genomes following the manufacturer’s instructions in the SensiFAST Probe No-ROX One-Step Kit (Bioline).

### Detection of HuNoV genome in tissues and blood

Small intestinal tissues were collected during necropsies, directly frozen in liquid nitrogen, and then stored in −80 °C freezer. 40 to 60 mg of frozen tissues were thawed at room temperature, washed in 1 ml PBS once, then homogenized in 0.2 ml TRIzol LS using Bullet Blender with 100 mg of 1.0 mm Zirconium Oxide beads (Next Advance). Total RNA from the homogenized tissues was isolated by adding 0.55 ml TRIzol LS. Blood was collected immediately after euthanasia, and 30% of ACD was added to prevent coagulation. Plasma and whole blood cells were separated by centrifugation at 2000*g* for 5 min. Plasma from 250 μl blood and whole blood cells from 50 μl blood for each pig were used for RNA isolation. 1 × 10^7^ MNC from ileum and spleen were used for RNA isolation. For these samples, RNA was isolated using 750 μl TRIzol LS following the manufacturer’s instructions, and the HuNoV genome copies were determined by TaqMan qRT-PCR as described above.

### Immunohistochemistry

Small intestinal tissues were collected upon euthanasia, fixed in 4% paraformaldehyde overnight, embedded in paraffin, sectioned at 5 μm, and placed on positively charged glass slides. Tissue slides were deparaffinized and rehydrated by washing in a graded ethanol series. For enzymatic antigen retrieval, slides were digested in 80 μg/ml proteinase K solution (Sigma Aldrich) for 30 min at 37 °C, followed by washing with tris-buffered saline (TBS). For blocking, slides were incubated in TBS containing 10% normal pig serum and 1% BSA for 2 h at room temperature. For IHC of duodenum and jejunum, slides were incubated with a goat anti-HuNoV GII.4 VLP polyclonal antibody diluted in TBS containing 1% BSA overnight at 4 °C, then washed with TBS containing 0.025% Triton X-100, and incubated with Alexa Fluor 488-labeled donkey anti-goat secondary antibody (A-11055; Thermo Fisher Scientific; 1:500) diluted in TBS containing 1% BSA for 1 h at room temperature. For IHC of ileum, mouse anti-CD79 (VP-C366; Vector Laboratories; 1:1000) and Alexa Fluor 546-labeled donkey anti-mouse secondary antibody (A10036; Thermo Fisher Scientific; 1:500) were also included in the two incubation steps above. Finally, slides were washed in TBS and mounted in Vectashield containing 4,6-diamidino-2-phenylindole (DAPI) for counterstaining cell nuclei (Vector Laboratories). Images were acquired on a Zeiss LSM 880 confocal laser scanning microscope in Fralin Imaging Center at Virginia Tech.

### Ileum Peyer’s patches histopathology

Sections of ileum were prepared as described above, and H&E staining was performed routinely. A pathologist was blinded to identification of the samples and evaluated the IPP histopathology using a light microscope with an ocular micrometer. To characterize the IPP sizes, muscularis mucosae to muscularis (mm to m) associated with gut associated lymphoid tissue (GALT), width of GALT, height and width of follicles were measured. For each parameter, 12 random locations including all pigs in each group were measured.

### Statistics

Pigs (male and female) were randomly divided to control group and *E. cloacae* group, pigs infected with HuNoV in each group were randomly assigned to be euthanized on PID3, PID7, or PID10. To assess clinical signs and virus shedding, pigs euthanized on PID10 contributed data to PID3 and PID7 subgroups, as did PID7 contribute data to PID3 subgroup. Statistical significance was determined with analyses specified in figure legends and table notes using GraphPad Prism 6.0 (GraphPad Software). *P* value < 0.05 was indicated as statistically significant.

## Additional Information

**How to cite this article**: Lei, S. *et al.* *Enterobacter cloacae* inhibits human norovirus infectivity in gnotobiotic pigs. *Sci. Rep.* **6**, 25017; doi: 10.1038/srep25017 (2016).

## Figures and Tables

**Table 1:** Incidence of clinical signs and fecal virus shedding in Gn pigs after HuNoV GII.4 2006b infection.

Group	Time	*n*	Diarrheaa,^b^			Virus shedding^b^		
			Pigs with diarrhea (%)*	Mean days to onset (SEM)**	Mean duration days (SEM)**	>Pigs shedding virus (%)*	Mean days to onset (SEM)**	Mean duration days (SEM)**
Control	PID3	9	4 (44%)	3.2 (0.3)	0.4 (0.2)	7 (78%)	2.1 (0.4)	1.4 (0.3)
*E. cloacae*		8	4 (50%)	3.0 (0.4)	0.7 (0.3)	6 (75%)	2.1 (0.4)	1.5 (0.4)
Control	PID7	6	6 (100%)	3.8 (0.7)	3.0 (0.4)	6 (100%)	2.3 (0.6)	4.3 (0.5)
*E. cloacae*		4	4 (100%)	4.3 (1.5)	2.0 (1.0)	4 (100%)	1.8 (0.3)	4.3 (0.5)
Control	PID10	3	3 (100%)	2.3 (0.3)	3.7 (0.3)	3 (100%)	1.3 (0.3)	6.7 (0.3)^**A**^
*E. cloacae*		4	4 (100%)	4.3 (1.5)	2.7 (0.9)	4 (100%)	1.8 (0.3)	5.5 (0.3)^**B**^

All pigs were inoculated with a HuNoV GII.4 2006b variant 092895 at 6 days of age. Daily rectal swabs were collected after inoculation to assess diarrhea and HuNoV shedding by Taqman qRT-PCR.

^a^Pigs with daily fecal scores of ≥2 were considered diarrheic. Fecal consistency was scored as follows: 0, solid; 1, semisolid; 2, pasty; 3, semiliquid; and 4, liquid.

^b^Fecal consistency score or virus shedding calculation included all the pigs in each group from PID1 to PID3, PID7, or PID10. If no diarrhea or virus shedding was observed, the days to onset were recorded as 1 day longer than each timepoint (4, 8, or 11) and the duration days were recorded as 0 for statistical analysis.

^*^Fisher’s exact test or

^**^Student’s *t*-test was used for statistical analysis. Groups with significant differences (*P* < 0.05) were indicated with letters A and B.
